# Biventricular repair using subvalvular techniques for unbalanced atrioventricular septal defect

**DOI:** 10.1093/icvts/ivad109

**Published:** 2023-07-14

**Authors:** Gen Shinohara, Shinichiro Oda, Satoshi Fujita, Akira Shiose

**Affiliations:** Department of Cardiovascular Surgery, Kyushu University Hospital, Fukuoka, Japan; Department of Cardiovascular Surgery, Kyushu University Hospital, Fukuoka, Japan; Department of Cardiovascular Surgery, Kyushu University Hospital, Fukuoka, Japan; Department of Cardiovascular Surgery, Kyushu University Hospital, Fukuoka, Japan

**Keywords:** Unbalanced atrioventricular septal defect, Biventricular repair, Artificial chordae reconstruction

## Abstract

We report the case of a 19-month-old girl with a right-dominant unbalanced atrioventricular septal defect and severe right-sided atrioventricular valve regurgitation who underwent biventricular repair using basal chordae resection, artificial chordae reconstruction and a left-sided atrioventricular valvuloplasty. At 14-month postoperative follow-up, the patient had minimal heart failure, gained weight and adapted to biventricular circulation.

## INTRODUCTION

An unbalanced atrioventricular septal defect is an atrioventricular canal with 2 ventricles, one being disproportionately small, and a candidate for biventricular repair (BVR). Recent reports on complementary objective measures for clinical diagnosis [1] and BVR cohorts [2] have provided insights into treatment strategies. We report a case of successful BVR using subvalvular techniques.

## CASE REPORT

A 19-month-old girl was referred to our hospital after birth with a heart murmur and was diagnosed with right-dominant uAVSD (RDuAVSD), double-outlet right ventricle (RV) and severe right-sided atrioventricular valve (AVV) regurgitation (AVVR) on echocardiography. No extracardiac genetic or structural abnormalities were observed. On day 28, the patient underwent main pulmonary artery banding and gained weight.

**Figure 1:**
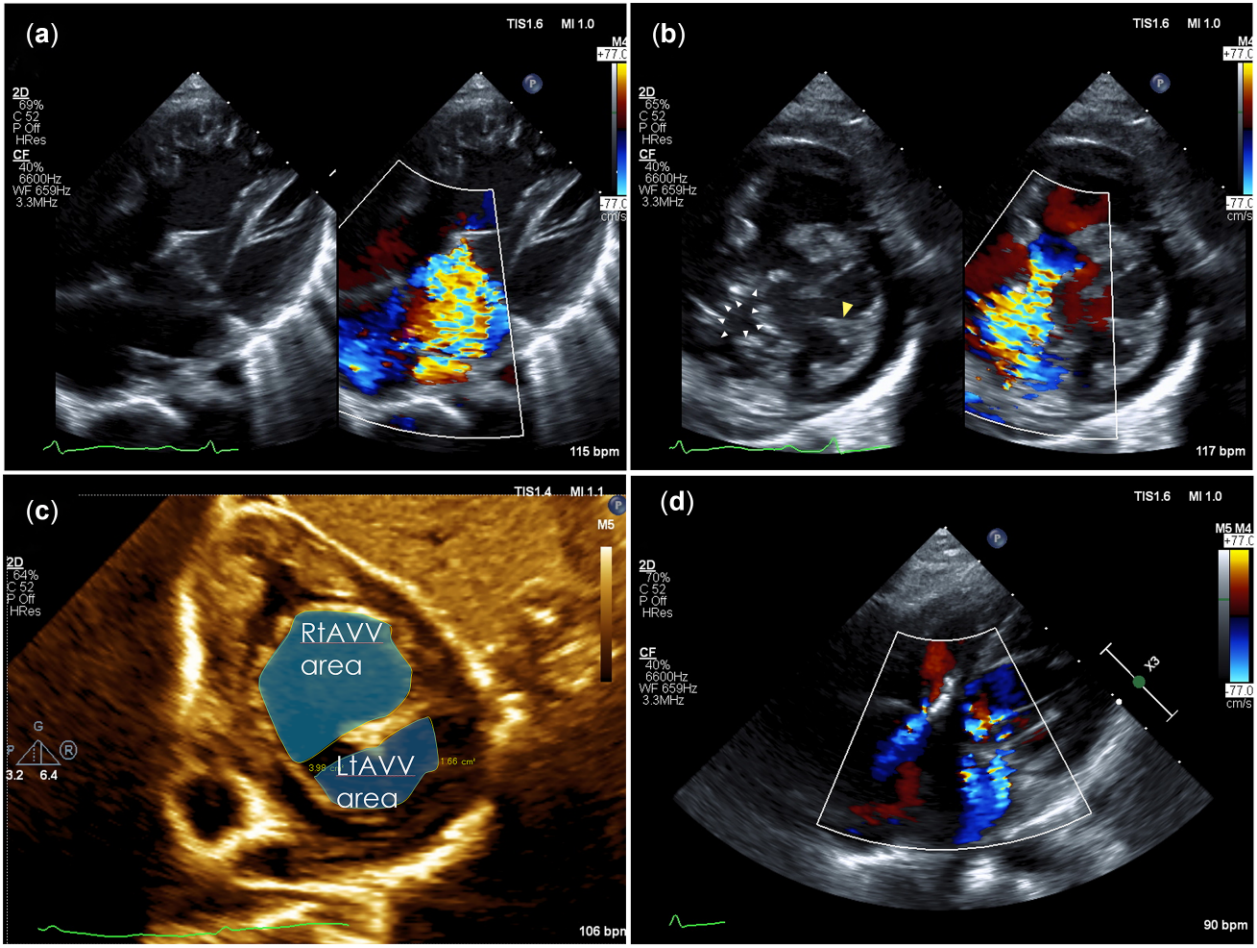
Preoperative echocardiography revealed severe right atrioventricular valve (AVV) regurgitation (annulus size 123% of normal) (**a**). Extensive gap between leaflets (**b**, white arrowheads). Marked hypoplasia of the left mural leaflet and single papillary muscle (yellow arrowhead) in the left atrioventricular valve (annulus size, 96% and 59% of normal on parasternal long-axis, four-chamber view, respectively). The modified AVV index was 0.29 (**c**). Echocardiography at 14 months postoperatively showed moderate left- and right-sided atrioventricular valve regurgitation (**d**).

**Figure 2:**
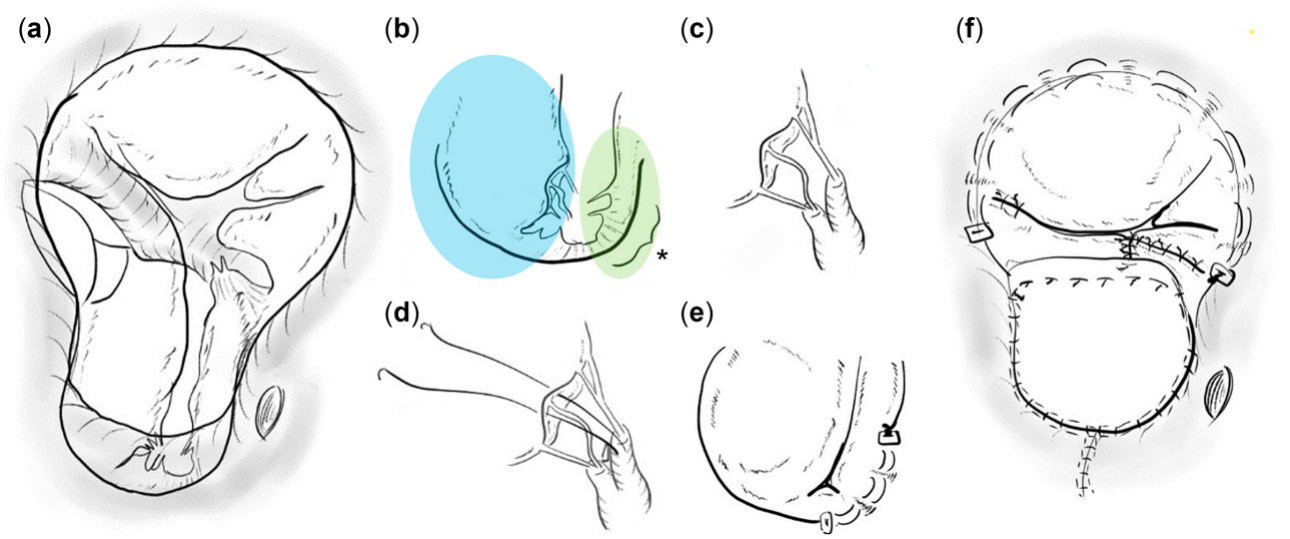
Interventricular communication without posterior extension and hypoplasia of the left-sided posterior bridging leaflet (PBL) continuously from the left mural leaflet (**a**). Atrioventricular septal defect repair was performed using the 2-patch technique with the left cleft untreated and opened, maintaining the left atrioventricular valve (AVV) orifice area without interatrial fenestration. Right AVV repair using semi-circular annuloplasty, cleft closure, edge-to-edge leaflet repair (**f**) and concomitant pulmonary artery debanding. Transoesophageal echocardiography showed moderate left AVV regurgitation (AVVR). During additional repair under second cardiac arrest, left AVV revealed restricted tenting of the PBL to the left mural leaflet (**b**). Prolapse of the anterior bridging leaflet due to elongated chordae attached to the single papillary muscle (**c**). Basal chordal resection of the PBL, artificial chordae reconstruction in the anterior bridging leaflet (**d**). Partial annuloplasty in the PBL (valve orifice 12 mm/75% of normal) (**e**). Subsequent transoesophageal echocardiography confirmed trivial left AVVR (inflow 0.85 m/s) and right AVVR (inflow 0.75 m/s) with 10mmHg of left atrial pressure, repair was completed.

The height and weight of the patient were 71 cm and 7.9 kg, respectively. Cardiac catheterization revealed a ratio of pulmonary to systemic flow (Qp/Qs) of 1.58, a ratio of pulmonary to systemic pressure (Pp/Ps) of 0.17 and pulmonary vascular resistance (RpI) of 1.6 Wood unit m^2^. Ventriculography showed RV and left ventricular (LV) end-diastolic volume of 291% and 102% of the normal value, respectively; right-sided AVVR of 3° and overriding aorta of >50%. Echocardiography results are shown in Fig. 1(a-c). The patient underwent a BVR with atrioventricular valvuloplasty. The intraoperative findings are shown in Fig. 2 and Video 1.

The patient was weaned from the ventilator on postoperative day 4 and discharged on day 43. Subsequently, heart failure symptoms reduced, and weight was gained favourably.

Echocardiography at 14 months postoperatively showed moderate left- and right-sided AVVR without ventricular enlargement (Fig. 1d), tricuspid regurgitant pressure gradient of 32 mmHg and no LV outflow tract or right ventricular outflow tract lesions.

## DISCUSSION

A quantitative-based definition for uAVSD has recently been proposed for scientific decision-making. Here, the modified AVV index was 0.29, within the range of RDuAVSD for BVR candidates (0.19–0.39) [1, 3]. In contrast, RV/LV angle [4] and LV inflow index [5], other predictors of treatment strategy, were 115 and 0.49, respectively. These were borderline values related to single-ventricular palliation (SVP) (114 and 0.5, respectively). Here, while modified AVV index provided the expectation of a valve orifice area, additional valvuloplasty can achieve acceptable left AVV function and BVR.

AVSD repair without a left-sided AVV procedure resulted in moderate AVVR requiring valvuloplasty, including subvalvular procedures. During the biventricular conversion from SVP for RDuAVSD, the subvalvular procedures include secondary chordal resection, accessory LV outflow tract tissue/muscle resection, chordal shortening/artificial chordae and delamination/splitting of papillary muscle [2].

We combined these common techniques in a single case. The procedures require secondary chordae resection for tethering of the hypoplastic left mural and posterior bridging leaflets, and artificial chordae for chordae elongation of the largely developed anterior bridging leaflet. Thus, valvuloplasty procedures for asymmetric structural developmental abnormalities were unique to uAVSD and should be considered when establishing and maintaining BVR. While BVR is beneficial to avoid SVP in uAVSD, surgical reinterventions for the left AVV are common [2].

## CONCLUSION

AVV repair, including subvalvular procedures, can be the key to achieve BVR in uAVSD.

## Data Availability

The data underlying this article cannot be shared publicly due to for personal information protection of individual that participated in the Case report. The data will be shared on reasonable request to the corresponding author.
